# Working with C. S. Sherrington, 1918–24

**DOI:** 10.1098/rsnr.2007.0037

**Published:** 2008-01-10

**Authors:** E.M. Tansey

**Affiliations:** Wellcome Trust Centre for the History of Medicine, University College London183 Euston Road, London NW1 2BE, UK

Sir Charles Sherrington FRS (1857–1952) was one of the most notable neurophysiologists of the twentieth century.^1^ After studies in Cambridge and London, he became a lecturer in physiology at St Thomas's Hospital, London, then Professor of Physiology at Liverpool in 1895, and then Waynflete Professor of Physiology at Oxford (1913–35). His career focused on the structure and function of the nervous system, exemplified by his analysis of the reflex arc, summarized in *The integrative action of the nervous system* (1906), a significant landmark in modern neurophysiology.^2^ He described the reciprocal innervation of antagonistic muscles, by which the activity of one set of excited muscles is integrated with another set of inhibited muscles; he coined the word ‘synapse’ to describe the junction between nerve cells; he studied numerous sense organs; and he mapped the motor areas of the cerebral cortex of mammals. He was elected FRS in 1893 and knighted in 1922. He was President of the Royal Society from 1920 to 1925 and shared the 1932 Nobel Prize for Physiology or Medicine with Lord Adrian FRS.

This interview with Mr T. J. Surman was conducted in Cardiff in June 1996, transcribed and edited by E. M. (Tilli) Tansey, and funded by the Physiological Society. Some of the interview has been previously published in the Physiological Society's newsletter and is reproduced with permission.^3^ The original tape and transcript will be deposited in the Physiological Society Archives, Archives and Manuscripts, The Wellcome Library, London.

Tansey: When did you start with Charles Sherrington?

Surman: 1918, just as the First World War was finishing, I was 14 at the time. I'm an Oxford boy, born and brought up there. My father was away as a soldier in France, and they used to have volunteer ladies calling on the wives of the soldiers, and it was the lady who visited us who said, ‘You're leaving school aren't you? I'll ask my friend Professor Sherrington if he wants a lab boy.’ That was how I started there.

Tansey: Where was the lab then?

Surman: In the University Museum—there's the big museum in the front, and all the departments were behind. When I first went there it was Physiological and Chemical Physiology, then they made a separate Professor of Chemical Physiology and called it Biochemistry. The first professor was Benjamin Moore.^4^ What happened was, there used to be a visiting professor, Professor Moore of Liverpool, who had a friend, Mr Whitley, who gave the money to create the Chair of Biochemistry. Moore got the chair and was first Professor of Biochemistry in Oxford.^5^

Tansey: You were in Physiology, not Chemical Physiology?

Surman: Yes.

Tansey: What did you do? What were your duties?

Surman: In research, helping the professor with his experiments and with classwork.

Tansey: How many people were there?

Surman: There was the professor, one senior lecturer, I think, and another couple—every year two of the young men who had passed the honours exams the previous year came as demonstrators.

Tansey: What about technicians?

Surman: There was George Cox the chief technician and two or three of us lab boys—not much really.^6^

Tansey: Who taught you what you needed to know?

Surman: You just picked it up as you went along—George Cox was supposed to help. Cox had been with the professor right from the time he had been in St Thomas's as an assistant lecturer, when George Cox had been his lab boy. He'd been with him all the time he'd been professor in Liverpool, and had travelled all around with Sherrington, and finished up in Oxford with him, was with him virtually all his life.

Tansey: How did you help Sherrington with his experiments?

Surman: I assisted at the operating table; when I was there he was working mainly on proprioceptive reflexes in cats. I was there for six years and during that time he used one dog; he wanted to do some cooling experiments, and when he finished he gave the dog to someone as a pet. He didn't mind using cats, but he loved dogs too much, and didn't like using dogs.

We had a mammalian class there with six or seven cats every week, on a Tuesday; they mainly came in from Liverpool, a chap there who used to supply us with cats.

Tansey: How many students were there then?

Surman: When I started there were only a few, not more than 10–12, but when I left there were 30–40 there. The big influx came when the First World War finished and returning servicemen got grants.

Tansey: Was Graham Brown in Oxford when you were there?

Surman: Brown was never in Oxford, although he was associated with Sherrington. He was in Liverpool with Sherrington and used to come down to Oxford to the professor quite a bit, but he was never in Oxford. He only came down temporarily there.^7^

Tansey: Was this how you knew him?

Surman: Sherrington used to have foreigners and all sorts to work with him, usually for six months or so. I worked with them all. That's how I met Graham Brown. When I came to Cardiff we had a couple come to work with him. The big trouble with him [Brown] was he lost interest in physiology when he got interested in mountaineering.^8^

Tansey: When was it you came to Cardiff to work with Graham Brown? 9

Surman: 1924—I was in Oxford just over six years.

Tansey: What kind of a man was Sherrington?

Surman: He was nice to work for, but very quick tempered, but he'd tell you off but never kept it going. You were in for a real good telling off if you did something wrong.

Tansey: What might you do wrong?

Surman: He was very fussy about apparatus—if you left apparatus out in the open. If he happened to see something on the table uncovered, he'd give you a real good lacing down. If you used a piece of apparatus, when you were finished you had to put it back in its place, and if he saw it lying about, he'd go mad. I liked him very, very much; I got on extremely well with him. I did most of my work with him when he was working with Liddell.^10^ He was an easy man to get on with.

Tansey: Did you realize what an important man he would become?

Surman: Oh no—he was just plain Professor Sherrington. I remember he got the knighthood, Cox saying, ‘we'll have to get that name on the door changed to Sir Charles Sherrington’, and he said, ‘I'm still Professor Sherrington, aren't I?’ He was a busy man. He'd come in in the morning: ‘Right, Surman, we're doing an experiment now’; we'd work until three o'clock, he'd pack up and put on his hat and then down to the station, off to London to be the President of the Royal Society.

Tansey: Did he do an experiment every day?

Surman: Oh no, one or two a week, but he ran the mammalian class. Never missed it. Never went outside the door. And ran the frog class one afternoon a week. He had damn good help in George Cox. George Cox ran that building; no doubt about that.

Tansey: Did you learn a lot from George Cox?

Surman: Oh yes, George Cox was a master at his job. In fact I think I knew more than him at the end. He was really good.

Tansey: Did you help Sherrington with his own research work?

Surman: Oh yes, with the proprioceptive work, and he always took the mammalian class. Sometimes it was five cats and two rabbits. That was his main job—he was very proud of that mammalian class.^11^

Tansey: [Opening a copy of the book] You must have contributed to some of this?

Surman: He wrote that whilst I was there [in Oxford]. Mind you, I was only a young fellow; I didn't understand at the time half of it. I was busy learning it myself.

Tansey: Did Sherrington explain it to you?

Surman: Oh yes.

Tansey: Did you have to smoke all the kymograph papers?

Surman: Yes, that was my job. The professor was very good at the drawings.^12^

Tansey: What did you do for the classes?

Surman: Lay out all the instruments and equipment, help out the students, because after a bit I knew it all inside out; I was a demonstrator as well really. I really enjoyed that mammalian class. There were four big, double kymographs in there to start with, and I had to do all the [kymograph] papers, and each one had a mercury manometer—they were a blooming nuisance—the numbers of time the students would knock the mercury over.^13^

Tansey: And you had to recalibrate them?

Surman: Yes—they were the bane of my life, those manometers. But I loved that class. It was only one day a week but it took the rest of the blooming week to get it ready. I used to have a few rows with George Cox about the class. I used to say to him, ‘Look, if a student uses a syringe and puts it down, and I miss it when I clear up, it sticks. They have to supply all their own instruments, why don't they supply their own damn syringe?’^14^ He'd say, ‘Never you mind that—it's none of your business.’ Anyway next term they had to supply their own syringe, and you never saw one lying about. It was a real problem—you can't get the syringes unblocked then—you'd boil them and all sorts.

Tansey: Did you have individual circulation pumps?

Surman: There was a big one downstairs and a smaller one upstairs: we had Brodies [Brodie respiration pumps]. Every table got respiration; for all the decerebrate cats you had to keep them on circulation.^15^

Tansey: Did you prepare the cats? Did you do the decerebrations?

Surman: I never had a licence at Oxford, so I couldn't.^16^ I didn't have a university degree, which you needed then to get a licence. I got one when I came down here [Cardiff]. I anaesthetized the cats; it's quite a skill, getting them deep enough.

Tansey: Was there a problem with blood clotting?

Surman: They used a solution—not a well-known thing; I used to think it was the wrong thing. The good one [heparin] was very expensive. I was also going around helping to change cannulas. They wouldn't have been what you had—with a T-piece. They just had straight cannulas; you couldn't wash it out, so you had to take it out, put a loop on the artery, clean it out, and put it back in again.

Tansey: Were the kymographs motorized?

Surman: We had motors on each kymograph eventually. When I started there was a big belt drive down in the basement that drove them all. I remember one time being told off really badly; we were doing a demonstration. Daly was the chap—I never liked him afterwards—told me off in front of all the students. We were doing a demonstration, and came to mark the kymograph trace, and there was no motor on it, [he] told me off for ‘not doing your job properly’ and later some bloke, a Dr Fry, walked in with the motor: ‘Sorry, I had to borrow your motor’—he'd taken it off just before. Daly apologized to me then, but not in front of the students. That did annoy me. Because if I did something as a demonstration I did it properly.^17^

Tansey: What was the frog class?

Surman: Mainly nerve stimulation; they did it all themselves. All they had to do was kill a frog and get on with it. There was very little to do in that class. During the final exams in one of the frog classes there was one chap I liked very much, and in the exam the frog class was a long room, tables coming out from one side, and for the exams we put a screen between the tables, and I used to walk up and down the outside. This chap was in a hell of a mess, and when I went by, I never stopped, I just put my two fingers on the induction coil as to where he should connect up. The examiner, a chap called Flack, said, ‘You just showed that man how to connect his coil.’ I had to admit I did, and I was sent out to get George Cox to send in another technician.^18^ He chucked me out.

Tansey: What happened to the student? Did he pass?

Surman: Oh yes, we didn't get many failures in that class.

Tansey: Did you have a common room? What did you do at lunch time?

Surman: There was no refectory or anything. I'd often go into the town, into Oxford Market to one of the little stalls there.

Tansey: What about the scientists? Where did they eat?

Surman: Sherrington ate in his own room. They were very short of room there. George Cox had his own room, and I used to spend a lot of time in his room. But there were few private rooms.

Tansey: Did you have contact with technicians from other departments?

Surman: Not an awful lot—I knew the technicians in other departments, in anatomy and pathology, Edwin Weale and his brother, technician and chief technician in pathology. And then they moved to a new building, down South Parks Road, and I never saw anything of them.

Tansey: Was there any kind of technicians' club?

Surman: Yes and no—we'd get together sometimes for a big dinner, at the Randolph Hotel and places like that. There was talk of a club but it never came to anything.

Tansey: Cambridge had a technicians' club, and they had a cricket team and such things.

Surman: In Oxford they talked about it for donkey's years but nothing materialized. They had bags of scope for it. They had a cricket team from the Museum—all the departments. I wouldn't mind being back there now if I was young enough.

Tansey: You'd find it very different.

Surman: Oh yes, no doubt. Of course I left before the new building—I've been in it, mind, but never worked there. The old one was a lovely building—very compact. Downstairs there was the mammalian class and the frog class, and lots of small rooms, and upstairs there was chemistry, which was biochemistry and histology. There was a huge room for histology.

Tansey: Did you ever do histology?

Surman: Not much—I did a little. Chap named Giles was there. Harry Carleton was the lecturer there. I can picture that department upstairs—the washing-up room and the store room. There were two flights of stairs—one each end. And when you had exams and students it was very useful. When you had a changeover one lot would go down one staircase whilst the next lot came up the other, so they didn't meet. Harry Carleton was a hell of a nice chap but had an awful stammer.

Tansey: Did they have separate heads of department at the beginning?

Surman: I suppose Sherrington was head of it all. But I can't remember, to be honest.

Tansey: How much were you paid?

Surman: 7s. 6d. a week to start with, and when I came to Cardiff first of all I was getting 30s. a week. I came as senior technician; chief technicians hadn't been made up in Cardiff then—they were very slow in making them up—I was the first, but that was some years after I got here. When I came there were two seniors. I'd been junior technician in Oxford with George Cox.

Tansey: Did you ask Sherrington to write a reference for you for the Cardiff job?

Surman: No, often thought I should have done—would have got me a job anywhere, wouldn't it? Something that makes me laugh is he used to call me ‘Boy’, but when I'd been there a couple of years he would call me ‘Boy’ and then say, ‘Sorry, Surman, I must remember to call you by your name’, and then he would call me ‘Boy’ again.

Tansey: When you came to Graham Brown in Cardiff, did he invite you? Did you apply for the job?

Surman: He was a big pal of Sherrington's, and he needed a technician. He'd brought Bonus [?] with him from Manchester, a one-legged technician. I never knew him; he was sacked because he always went away when Graham Brown went away. Graham Brown asked George Cox if there was a trained technician who'd go to Cardiff. I never applied, never had any intention of coming here. Whether I did the right thing or not? Actually I didn't. I should have stayed, as soon after I left George Cox retired and I could have had his job. You never know whether you're doing the right thing, but I've enjoyed my life down here, but I would have enjoyed staying in Oxford.^19^

Tansey: What was it like when you got to Cardiff?

Surman: I didn't like it a bit when I got here. It was very snobbish. The step between technicians and lecturers was very great. Oxford had been a lovely place. Dr Liddell, who became professor there after Sherrington, would come by and sit on the desk and chat whilst I was getting the classes ready. You never got that in Cardiff: the technical staff were very separate from the teaching staff. After Graham Brown left, that all changed; Peterson came. To be perfectly honest I almost packed it in when I first came down—I was absolutely browned off with it; the atmosphere was so different from Oxford.

Tansey: What made you stick at it?

Surman: I didn't like the job, to start with. Graham Brown got me here and the day I came to Cardiff he went away for three months, climbing mountains. I was hanging around the department for three months doing nothing. I was bored to tears. I wouldn't have come if I'd known.

Tansey: You were quite young—20—to come as a senior technician?

Surman: I was probably the youngest senior in the country. There were three lecturers and each had a technician and a junior technician, so there were six altogether. I had two juniors with me because I also had charge of the animal house.

Tansey: When did you get a licence?

Surman: I can't remember precisely when. I remember the Home Office inspector at the time came round to see us, and Graham Brown said we'd like Surman to have a licence, and the Home Office Inspector said, ‘That depends on whether Surman wants a licence.’ I said, ‘Yes’, and he said, ‘That's it; you shall have one.’ The only thing was I couldn't get a recovery licence, all acute experiments with me.^20^

Tansey: Did you do class demonstrations?

Surman: I wasn't supposed to, but I did a lot. I was better than the rest; I did it more often. I enjoyed doing that.

Tansey: What were your main duties?

Surman: All the classwork—that is, prepare all the classes, and assist, and if anyone's partner didn't turn up, I'd help out.

Tansey: How many students?

Surman: Probably about 12 or so when I came, but 60 or so when I finished, but not all of them did honours; about 30 of them did honours.

Tansey: Did you do the same sort of experiments as in Oxford?

Surman: Yes, they all worked from ‘The Book’.^21^
